# Objective Assessment of Equine Locomotor Symmetry Using an Inertial Sensor System and Artificial Intelligence: A Comparative Study

**DOI:** 10.3390/ani14060921

**Published:** 2024-03-16

**Authors:** Natalie Calle-González, Chiara Maria Lo Feudo, Francesco Ferrucci, Francisco Requena, Luca Stucchi, Ana Muñoz

**Affiliations:** 1 Department of Animal Medicine and Surgery, School of Veterinary Medicine, University of Córdoba, 14004 Córdoba, Spain; pv1mujua@uco.es; 2 Equine Sport Medicine Center CEMEDE, School of Veterinary Medicine, University of Córdoba, 14004 Córdoba, Spain; v02redof@uco.es; 3 Equine Sports Medicine Laboratory “Franco Tradati”, Department of Veterinary Medicine and Animal Sciences, Università degli studi di Milano, 26900 Lodi, Italy; chiara.lofeudo@unimi.it (C.M.L.F.); francesco.ferrucci@unimi.it (F.F.); 4 Department of Cellular Biology, Physiology and Immunology, University of Córdoba, 14004 Córdoba, Spain; 5 Department of Veterinary Medicine, University of Sassari, 07100 Sassari, Italy; lstucchi@uniss.it

**Keywords:** artificial intelligence, gait analysis, horse, inertial measurement unit system, lameness, locomotion

## Abstract

**Simple Summary:**

Lameness is one of the main causes of poor performance in all equestrian disciplines. Traditionally, it is assessed by clinical assessment, using subjective numerical scales. Quantitative lameness assessment has gained popularity over the last years, aiding in the decision-making process. Recently, a marker-less artificial intelligence (AI) motion tracking system has been developed for lameness assessment, requiring less specialized equipment than traditional methods, along with reduced technical expertise and time-consuming procedures. Our aim was to compare this system with an inertial measurement unit system, as well as with clinical examination, to determine the level of agreement and accuracy of both systems, and their relation to visual examination. In our study, clinical examination detected locomotor asymmetries in accordance with both systems. A greater number of limbs were considered asymmetric by the AI motion tracking system. The highest level of agreement was observed for forelimb movement on a straight line and hard surface, and the lowest for pelvic movement on a straight line and soft surface, probably due to the difficulty in assessing hindlimb asymmetry. It would be interesting to measure locomotor asymmetries regularly in training and conditioning horses, as some degree of asymmetry may be clinically relevant.

**Abstract:**

In horses, quantitative assessment of gait parameters, as with the use of inertial measurement units (IMUs) systems, might help in the decision-making process. However, it requires financial investment, is time-consuming, and lacks accuracy if displaced. An innovative artificial intelligence marker-less motion tracking system (AI-MTS) may overcome these limitations in the field. Our aim was to compare the level of agreement and accuracy between both systems and visual clinical assessment. Twenty horses underwent locomotion analysis by visual assessment, IMUs, and AI-MTS systems, under the following conditions: straight hard (SH), straight soft (SS), left and right circle hard (LCH, RCH), and soft (LCS, RCS). A greater number of horses were considered sound by clinical examination, compared to those identified as symmetric by the two gait analysis systems. More limbs were considered asymmetric by the AI-MTS compared to IMUs, suggesting its greater sensitivity. The greatest agreement between the two systems was found for the difference between two minima in vertical head position in SH, while the lowest for the difference between two minima in vertical pelvis position in SS, reflecting the difficulties in assessing asymmetry of the hindlimbs. It is unknown what degree of asymmetry is clinically relevant, suggesting that more consistent use in training horses may help determine the thresholds for asymmetry. Some degree of asymmetry may be clinically relevant, suggesting its regular use in training horses.

## 1. Introduction

Lameness is a major cause of poor performance, with serious consequences for horses, owners, and veterinarians in terms of the welfare of the equine athlete, the expectations of the trainer/rider, and the loss of economic income associated with competition [1,2]. In a clinical setting, the assessment of lameness involves observing various characteristics of the horse’s movement at different gaits, particularly focusing on the trot, but not exclusively [3]. Various subjective numerical scales have been developed to standardize the severity of lameness for communication among clinicians and for inclusion in clinical records for future consultation [4]. The American Association of Equine Practitioners (AAEP) grading scale is one of the most widely used for quantifying lameness severity [4,5]. This scale ranges from 0, indicating soundness, to 5, representing maximum lameness with minimal weight bearing.

Several studies have documented that the agreement in clinical subjective assessment using standardized scoring between experienced clinicians is low for lameness of mild to moderate intensity (1-2/5 on the AAEP scale) [6], with higher agreement in the detection of the lame limb, but not for the degree of lameness. Inter- and intra-clinician agreement is acceptable to poor, being poorer for hindlimb lameness compared to forelimbs [7,8]. Perception, expectation bias, and the limited resolution of the human eye are the main causes of the moderate to low levels of inter-observer agreement, as well as poor intra-observer reliability in visual lameness examinations [9,10,11,12]. 

To overcome these problems, several inertial measurement unit (IMU) systems have been developed in recent years to aid in the decision-making process, particularly in cases of mild lameness, referred lameness, evaluation of improvements after a treatment or recovery period, or when conducting clinical research studies [13,14]. 

Most of these IMUs are based on the assessment of gait symmetry during trot. They provide quantitative data on vertical movement symmetry and vertical displacement over multiple trot strides. Sensors are usually attached to primary axial body segments with double-sided tape; the head for forelimb lameness, the pelvis for hindlimb lameness, and sometimes the withers for compensatory lameness [15,16,17]. The normal vertical displacement of these body segments is represented by a sinusoidal double wave curve for each stride. The highest position of the two peaks and the lowest position or valleys of this signal are analyzed to indicate the level of asymmetric loading of the right versus left limb during the midstance and push-off phases of the trotting stride [18]. Several publications have reported that IMUs are reliable tools for correctly identifying and localizing the limb with pain or dysfunction [19,20,21,22]. These sensors are considered accurate and precise because they provide measurement parameters that are consistent with subjective visual assessments of lameness, along with data collected from force plate analysis. They have proven to be sensitive to small changes in movement symmetry, as observed in horses with experimentally induced lameness [23,24,25,26,27,28]. 

However, the use of IMUs requires a substantial financial investment, and mounting the sensors on the horse’s body is time consuming and may lack accuracy if the sensors are displaced, which limits their use in a field setting [29,30,31,32]. Recently, an artificial intelligence (AI) marker-less motion tracking system (AI-MTS) has been developed (Sleip AI, Uppsala, Sweden) [33]. It is based on deep neural networks that process large amounts of data from computer algorithms previously trained by deep learning on the horse’s body for objective motion analysis. In particular, it tracks the movement of the head, pelvis, and hooves of trotting horses, both towards and away from the camera, to obtain measurement images [33,34,35,36]. This system allows its use with minimal technical training and no expensive equipment, allowing assessment from a smartphone camera video, which is easier and more practical in a clinical setting [37]. 

The current study was conducted with the aim of comparing IMUs with AI-MTS, analyzing the level of agreement and accuracy of both systems, in different conditions (straight line and circles) and over two different types of surfaces (soft and hard), in horses considered sound or mildly lame at clinical examination. 

## 2. Materials and Methods

### 2.1. Horse Population

A convenience sample of twenty horses of different ages, sexes, breeds, and uses, referred to the Equine Sports Medicine Center (CEMEDE) of the University of Cordoba (Spain) for performance assessment, were enrolled on a voluntary basis by their owners during a 2-week data collection period. Depending on the owners’ preference, the procedures were performed either at the riding stables where the horses were normally kept (8 horses) or at the facilities of the Equine Sports Medicine Center (12 horses). Requirements for the stables were the presence of a hard flat surface (asphalt), and a sand riding arena, the same conditions found at the CEMEDE center, where horses could be trotted in hand, in a straight line, and lunged in circles of 10 m diameter.

Enrolled horses first underwent a clinical lameness assessment while trotting in hand on a straight line and in circles, on a hard and soft surface. Trots were recorded with a smartphone for later analysis. Two experienced blinded operators (N.C-G.) and (C.M.L.F.), assigned a score of 0–5 based on the AAEP lameness grading system and recorded the lame limb(s). Only horses with clinical lameness ≤ 2/5 were included in the study.

A signed written informed consent for the inclusion of horses in the present study and the use of data for research purposes was obtained from all owners. The followed procedures and the results derived were explained to the owners. All procedures were performed in accordance with the Code of Good Veterinary Practice. No specific ethical approval was required for the present study, as all horses were presented for veterinary examination and the cases were considered as professional activities.

### 2.2. Locomotor Assessment

The horses underwent gait analysis using two different validated gait analysis technologies simultaneously: an inertial sensor-based gait analysis system, such as IMUs (Equigait Ltd., Chestnut, Herts, UK) [38,39], and a smartphone application based on a computer vision system AI-MTS (Sleip AI, Uppsala, Sweden) [33,34]. To allow comparison of data collected by the two systems and to maximize synchronization between them, the recordings were manually started and stopped at the same time by two different operators. To reduce variability between trials, the same operators (C.M.L.F. and N.C-G.) were responsible for recording and collecting data from all the horses. 

The locomotion analysis of the included horses was performed in the following assessment conditions: (1) straight hard (SH): trot in hand on a straight line on a hard surface; (2) straight soft (SS): trot in hand on a straight line on a soft surface; (3) left and right circle hard (LCH and RCH): trot in circle on the left and right hand on a hard surface; (4) left and right circle soft (LCS and RCS): trot in circle on the left and right hand on a soft surface.

During the straight line data collection, horses were trotted by a handler at least two times back and forth on a 30 m long straight line as requested by the AI system. Handlers trotted the horses at their preferred speed and were asked not to interfere with the horses’ head movements. After that, the horses were lunged in a left circle for at least 45 s, stopped, and then lunged in a right circle for at least 45 s within a 10 m diameter circle. 

#### 2.2.1. Locomotor Assessment with an Inertial Measurement Unit System 

Horses were equipped with five inertial sensors (MTw2, Xsens, Enschede, the Netherlands) attached at specific anatomical locations: the poll (located at the center of the highest part of the head, between the ears), the withers (located at the most prominent part, over the spinous process of T6), the tuber sacrale (situated at the highest point of the pelvis), and the right and left tuber coxae. All the sensors were wirelessly synchronized with a station (Awinda, Xsens) that received the data and transmitted them simultaneously at a sampling rate of 100 Hz per channel to a dedicated laptop with a pre-installed analysis software (Equigait, Ltd., Chestnut, Herts, UK). Data recording was manually initiated and stopped by the same operator (N.C-G.) for all horses. For each assessment condition, the following data provided by the software were considered in the present study:

- HD_min_: difference between the vertical minima reached by the head during left and right forelimbs stance, expressed in millimeters;
- HD_max_: difference between the vertical maxima reached by the head during left and right forelimb stance, expressed in millimeters;
- H-ROM: range of motion of the head, difference between minimum and maximum values reached by the head throughout the stride cycle, expressed in millimeters;
- PD_min_: difference between the vertical minima reached by the pelvis during left and right hindlimbs stance, expressed in millimeters;
- PD_max_: difference between the vertical maxima reached by the pelvis during left and right hindlimb stance, expressed in millimeters;
- P-ROM: range of motion of the pelvis, difference between minimum and maximum values reached by the pelvis throughout the stride cycle, expressed in millimeters;
- Number of recorded strides;
- Laterality of the asymmetry (towards the left or the right side).

Furthermore, to allow a direct comparison between horses of different sizes and with different ranges of movement, as well as with the results of the AI-MTS, the values of HD_min_, HD_max_, PD_min_, and PD_max_ were normalized to H-ROM and P-ROM. For this purpose, HD_min_ and HD_max_ were divided by the H-ROM and then multiplied by 0.4; PD_min_ and PD_max_ were divided by the P-ROM and then multiplied by 0.25 [26,27]. The values obtained were defined, respectively, as follows: MinDiff_head_, MaxDiff_head_, MinDiff_pelvis_, and MaxDiff_pelvis_.

Finally, the HD_min_ and PD_min_ data recorded during the SH assessment were additionally analyzed by applying published thresholds to discriminate between symmetric and asymmetric horses. Specifically, kinematic thresholds of 12 mm for HD_min_ and 6 mm for PD_min_ were used, as suggested by previous studies [31,32]. After applying these thresholds, horses were classified as “symmetric” or “asymmetric”, and the asymmetric limb(s) were recorded.

#### 2.2.2. Locomotor Assessment with an AI Marker-Less Motion Tracking System

Simultaneously with the IMUs recordings, gait analysis was performed using a computer vision-based smartphone application (Sleip AI, Uppsala, Sweden), downloaded onto an iPhone 11 Pro. For this purpose, the smartphone was placed on a tripod at a height of 160 cm in landscape mode and the trots were recorded in all the assessment conditions described above. The video streams were first saved locally and later uploaded to the software for data processing.

Data recording was manually started and stopped for all horses by the same operator (C.M.L.F.). For each assessment condition, the following data provided by the software were considered in the present study:

- MinDiff_head_: difference between two minima in vertical position of the head, during the right and left forelimbs halves of a stride, normalized to the head range of motion;
- MaxDiff_head_: difference between two maxima in vertical position of the head, during the right and left forelimbs halves of a stride, normalized to the head range of motion;
- MinDiff_pelvis_: difference between two minima in vertical position of the pelvis, during the right and left hindlimbs halves of a stride, normalized to the pelvis range of motion;
- MaxDiff_pelvis_: difference between two maxima in vertical position of the pelvis, during the right and left hindlimbs halves of a stride, normalized to the pelvis range of motion;
- Number of recorded forelimb and hindlimb strides (as the pelvis was visible only when the horse was trotting away from the camera, the number of hindlimb strides was lower than that of the forelimb strides);
- Laterality of the asymmetry and the presumed affected limb(s).

For both IMUs and AI-MTS, the values of MinDiff_head_, MaxDiff_head_, MinDiff_pelvis_, and MaxDiff_pelvis_ were multiplied by −1 if the presumed affected limb was on the left side, while positive values indicated that the presumed affected limb was on the right side [33,34].

Moreover, the severity of any asymmetry was defined as follows: 0.3–0.5 (very mild asymmetry), 0.6–0.9 (mild asymmetry), 1–1.4 (moderate asymmetry). No horses in the study population had severe asymmetry, as horses with clinical lameness grade ≥ 3/5 were excluded. Thresholds for asymmetry severity were chosen based on the results provided by the AI-MTS application to allow diagnostic comparison between systems [33,34]. 

### 2.3. Statistical Analysis

Data were collected in an electronic spreadsheet (Microsoft Excel, Redmond, WA, USA), and analyzed using a commercial statistical software package (GraphPad Prism 9.5.1 for MacOS; GraphPad Software, San Diego, CA, USA). All data were tested for normality using the Shapiro–Wilk test, and descriptive statistics were performed according to the data distribution. Data are presented as mean ± standard deviation if normally distributed, or as median and interquartile range (IQR) if not normally distributed. Statistical significance was set at *p* < 0.05.

The agreement between the locomotor parameters evaluating the impact or push-off phases of the stride, as detected by the two technologies used (IMUs and AI-MTS), was evaluated using the K Cohen coefficient in each assessment condition. K Cohen coefficient values of 0 indicated no agreement, 0–0.2 slight agreement, 0.21–0.4 fair agreement, 0.41–0.6 moderate agreement, 0.61–0.8 substantial agreement, and 0.81–1 almost perfect agreement. Bland–Altman analysis was used to evaluate the statistical agreement between the gait analysis systems for each locomotor parameter in each of the assessment conditions.

In addition, the locomotion parameters were assessed by comparing the absolute values (without arithmetic signs) of each locomotion parameter in each assessment condition measured by the two technologies, using the paired *t*-test (for normally distributed data) or the Wilcoxon test (for non-normally distributed data). The number of strides recorded by the two systems in each assessment condition was compared using a paired *t*-test. To evaluate the effect of different surfaces and trot direction on the locomotor parameters, the data were compared using one-way ANOVA and Tukey’s multiple comparison test (for normally distributed data) or the Friedman test and Dunn’s multiple comparison test (for non-normally distributed data). In these tests, the absolute values were used for comparison, since the goal was to compare asymmetry regardless of laterality.

## 3. Results

### 3.1. Horse Population

The study population consisted of 20 horses aged between 4 and 24 years (median 9, IQR 8–15 years), including 15 geldings, 4 mares, and 1 stallion. The horses belonged to different breeds, including nine mixed breeds, five Spanish purebred horses, three Arabians, one pony, one Quarter Horse and one KWPN. Regarding horses’ use, ten were recreational horses, five were used for Spanish cowboy dressage competitions, three for endurance, and two for dressage. The results of the clinical examination and the symmetry data provided by the two locomotor analysis systems are shown in Table 1. 

The number of animals considered sound/symmetrical by clinical examination was nine; by IMUs, when using the HD_min_ and PD_min_ cut-off values, six; when using the severity cut-off values, zero; and by AI-MTS, one. Complete agreement between the three scoring systems (i.e., clinical examination, IMUs and AI-MTS) regarding the affected limb(s) occurred in only four horses. In all the horses that were considered lame on clinical examination, on the straight line and on a hard surface, IMUs and IA-MTS agreed on at least one of the affected limb(s) detected by clinical examination. However, both locomotor assessment systems detected more asymmetric limbs than those detected by clinical examination.

### 3.2. Agreement between Inertial Measurement Unit System and AI Marker-Less Motion Tracking System

Cohen’s coefficients of agreement between the two gait analysis systems (IMUs and AI-MTS) in the different assessment conditions (SH, SS, RCH, RCS, LCH, and LCS) are shown in Table 2 for the four locomotor parameters considered, (MinDiff_head_; MaxDiff_head_; MinDiff_pelvis_; MaxDiff_pelvis_). In none of the assessment conditions was Cohen’s coefficient greater than or equal to 0.81, which was indicative of almost perfect agreement. 

Substantial agreement between IMUs and AI-MTS was found for: MinDiff_head_ in SH, SS, and RCH; MaxDiff_head_ in RCH and RCS; and MaxDiff_pelvis_ in LCS, RCH, and RCS. The lowest agreement coefficients were found for MinDiff_pelvis_ in all the assessment conditions (Table 2).

**Figure 1:**
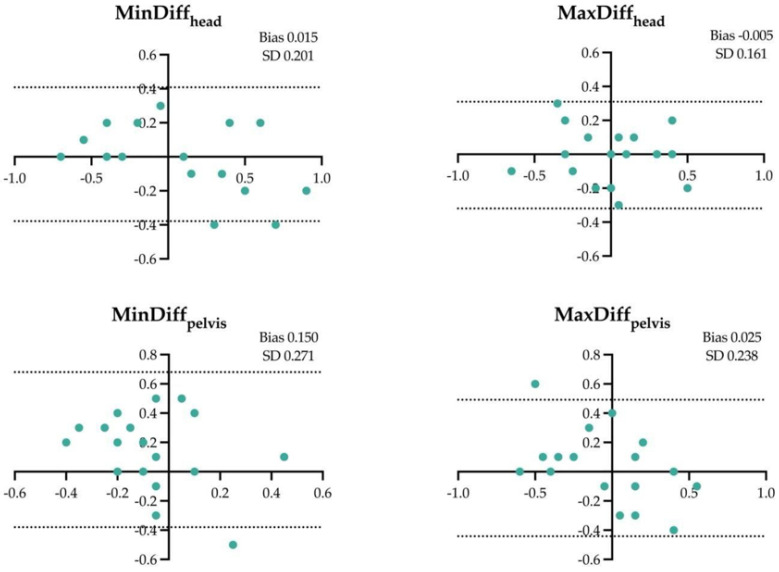
Difference between the locomotor parameters measured by inertial measurement unit (IMU) system and artificial intelligence marker-less motion tracking system (AI-MTS) (y axis) as a function of average value of both systems (x axis) for each straight hard (SH) assessment in the 20 horses included in the study. The upper and lower limits of agreement are illustrated by the dotted lines.

**Figure 2:**
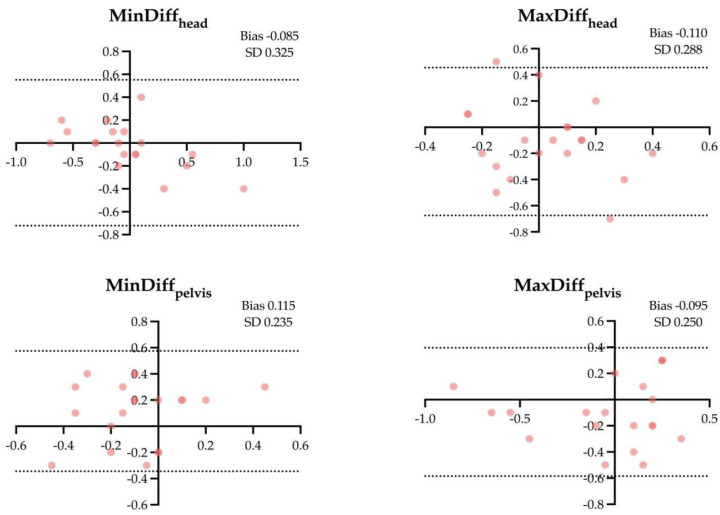
Difference between the locomotor parameters measured by inertial measurement unit (IMU) system and artificial intelligence marker-less motion tracking system (AI-MTS) (y axis) as a function of average value of both systems (x axis) for each straight soft (SH) assessment in the 20 horses included in the study. The upper and lower limits of agreement are illustrated by the dotted lines.

**Figure 3:**
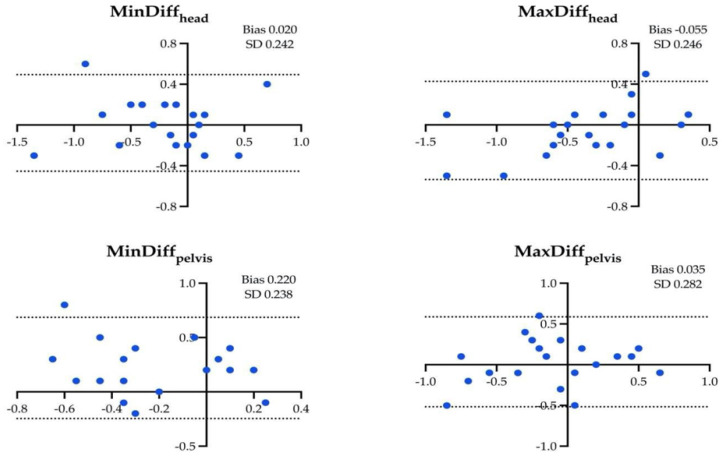
Difference between the locomotor parameters measured by inertial measurement unit (IMU) system and artificial intelligence marker-less motion tracking system (AI-MTS) (y axis) as a function of average value of both systems (x axis) for each left circle hard (LCH) assessment in the 20 horses included in the study. The upper and lower limits of agreement are illustrated by the dotted lines.

**Figure 4:**
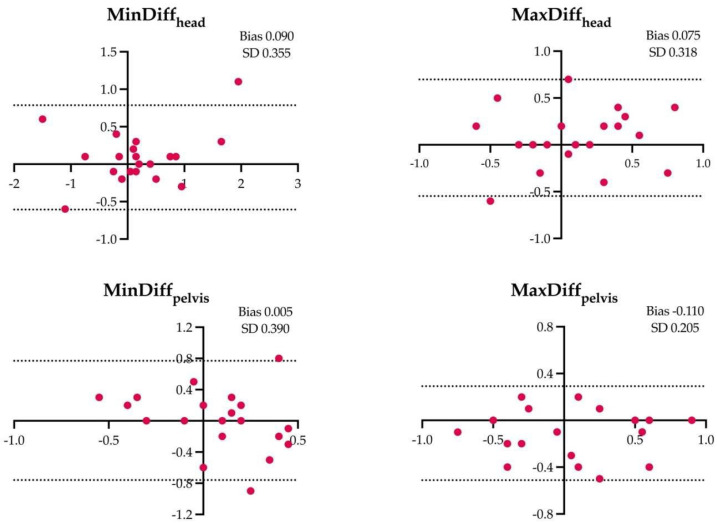
Difference between the locomotor parameters measured by inertial measurement unit (IMU) system and artificial intelligence marker-less motion tracking system (AI-MTS) (y axis) as a function of average value of both systems (x axis) for each right circle hard (RCH) assessment in the 20 horses included in the study. The upper and lower limits of agreement are illustrated by the dotted lines.

**Figure 5:**
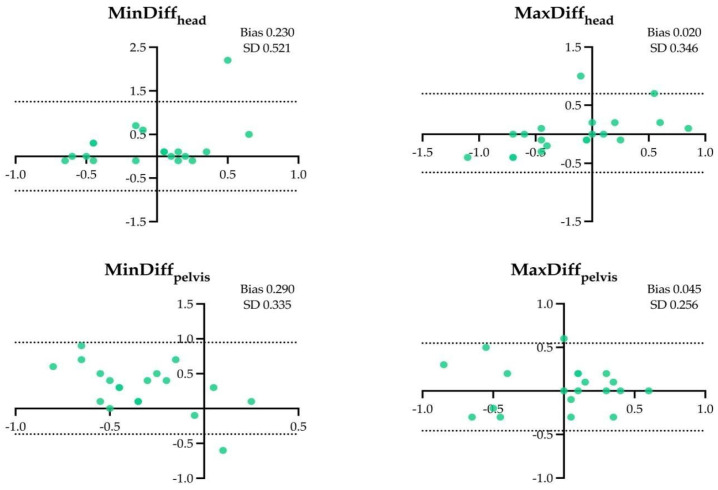
Difference between the locomotor parameters measured by inertial measurement unit (IMU) system and artificial intelligence marker-less motion tracking system (AI-MTS) (y axis) as a function of average value of both systems (x axis) for each left circle soft (LCS) assessment in the 20 horses included in the study. The upper and lower limits of agreement are illustrated by the dotted lines.

**Figure 6:**
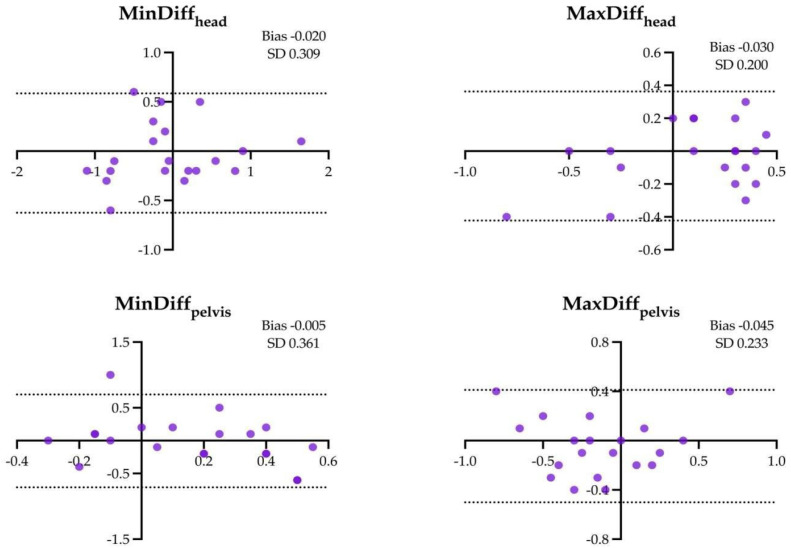
Difference between the locomotor parameters measured by inertial measurement unit (IMU) system and artificial intelligence marker-less motion tracking system (AI-MTS) (y axis) as a function of average value of both systems (x axis) for each right circle soft (RCS) assessment in the 20 horses included in the study. The upper and lower limits of agreement are illustrated by the dotted lines.

The results of the Bland–Altman analysis for the four locomotor parameters and for the different assessment conditions are shown in Figure 1, Figure 2, Figure 3, Figure 4, Figure 5 and Figure 6.

### 3.3. Significant Differences in the Locomotor Parameters between Inertial Measurement Unit System and AI Marker-Less Motion Tracking System

**Figure 7:**
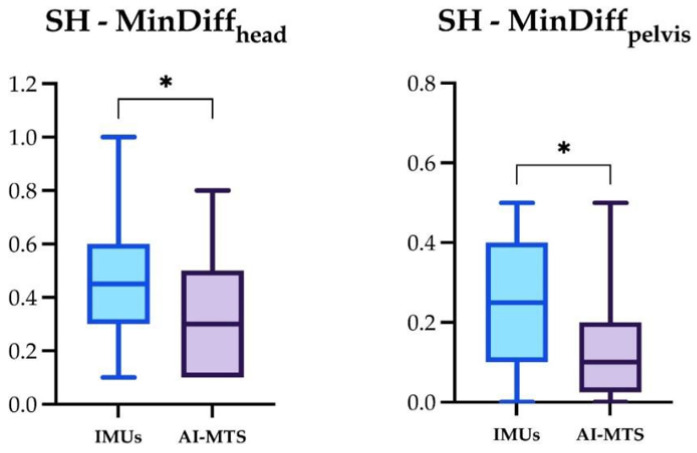
Box and whiskers plot showing median, interquartile range, and total range of the MinDiff_head_ and MinDiff_pelvis_ measured by the two gait analysis systems (IMUs and AI-MTS) during the straight hard (SH) assessments. Statistical significance was set as * (*p* < 0.05).

**Figure 8:**
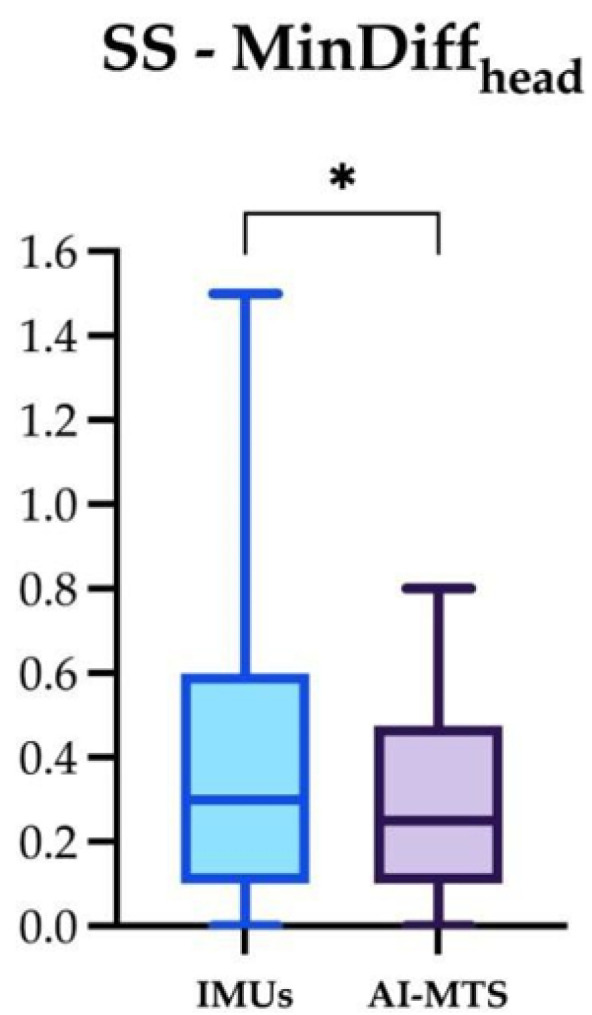
Box and whiskers plot showing median, interquartile range, and total range of the MinDiff_head_ measured by the two gait analysis systems (IMUs and AI-MTS) during the straight soft (SS) assessment. Statistical significance was set as * (*p* < 0.05).

**Figure 9:**
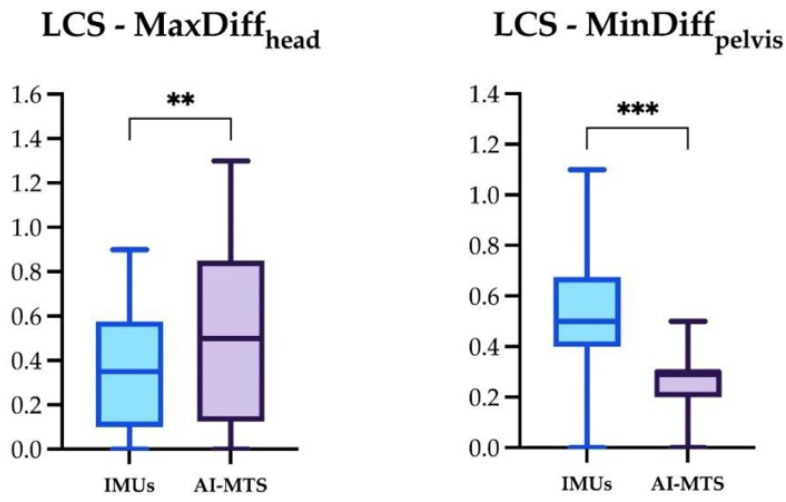
Box and whiskers plot showing median, interquartile range, and total range of the MinDiff_head_ and MinDiff_pelvis_ measured by the two gait analysis systems (IMUs and AI-MTS) during the left circle soft (LCS) assessment. Statistical significance was set as ** (*p* < 0.01) and *** (*p* < 0.001).

The significant differences for the four locomotor parameters using IMUs and AI-MTS are presented in Figure 7, Figure 8 and Figure 9 (only significant differences are shown). The following significant differences in locomotor parameters were found when comparing the two technologies. MinDiff_head_ values were significantly greater with IMUs compared to AI-MTS in SH and SS. Likewise, MinDiff_pelvis_ values were greater with IMUs in SH and LCS. On the contrary, higher MaxDiff_head_ values were found for AI-MTS in LCS (Figure 7, Figure 8 and Figure 9). 

The mean number of valid strides recorded by the IMUs and IA-MTS in the different assessment conditions are shown in Table 3. The number of recorded forelimb strides was significantly higher for the AI-MTS compared to the IMUs in SH, LCH, LCS, RCH, and RCS conditions. Conversely, the number of hindlimb strides was higher for the IMUs compared to the AI-MTS in the SS assessment, and lower in LCH and RCH conditions.

### 3.4. Effect of the Different Assessment Conditions

**Figure 10:**
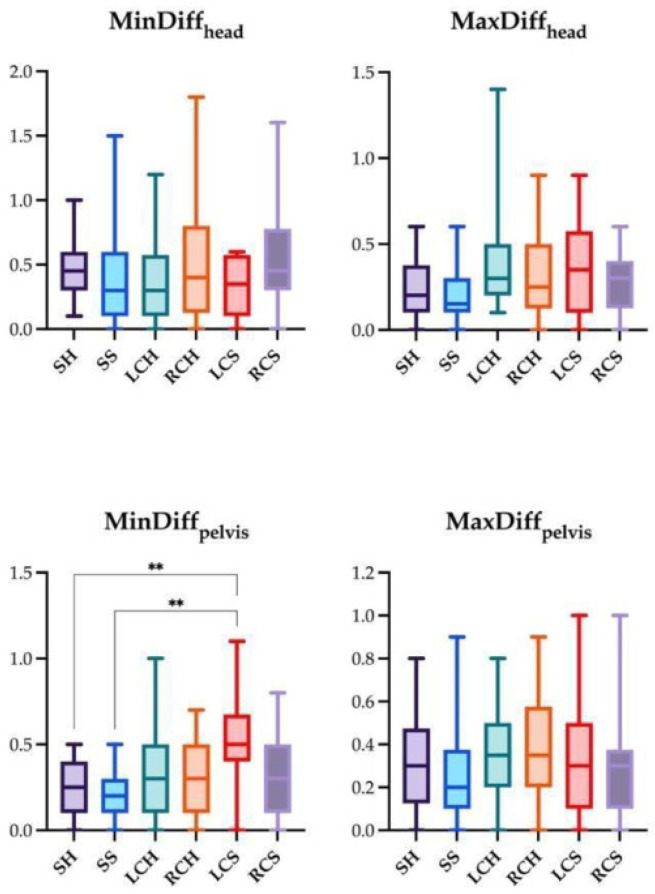
Box and whiskers plot showing median, interquartile range, and total range of the locomotion parameters measured by the inertial measurement unit (IMU) system in the following trotting conditions: straight hard (SH), straight soft (SS), left and right circle hard (LCH and RCH), left and right circle soft (LCS and RCS). Statistical significance is shown as ** (*p* < 0.01).

**Figure 11:**
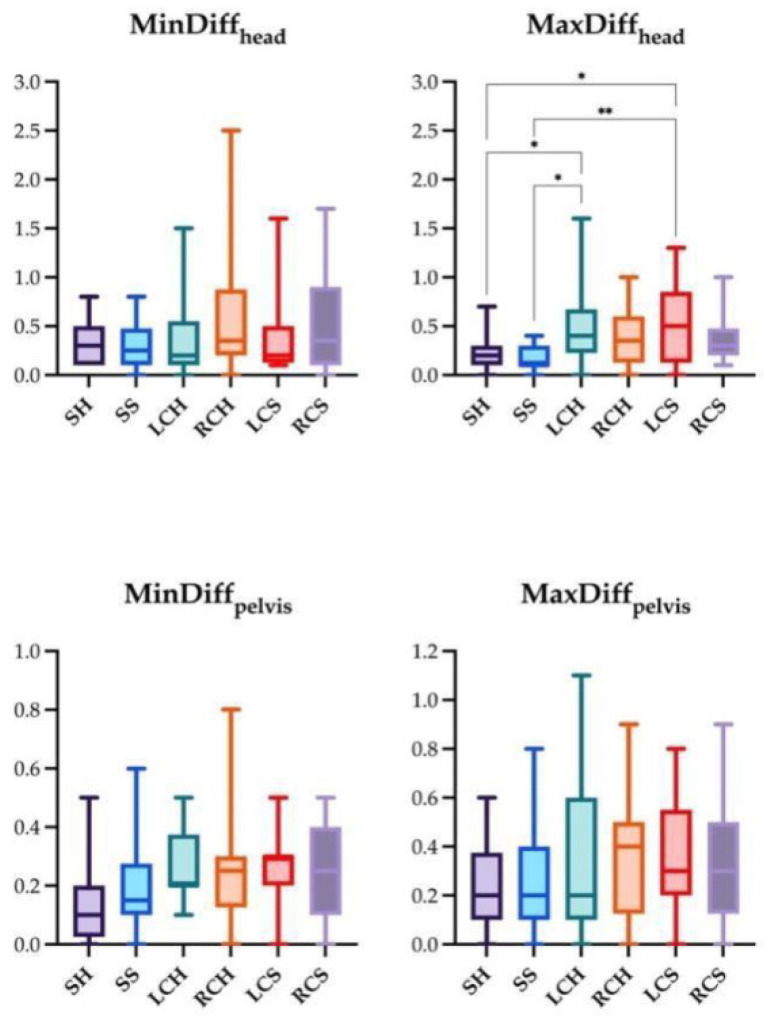
Box and whiskers plot showing median, interquartile range, and total range of the locomotion parameters measured by artificial intelligence marker-less motion tracking system (AI-MTS) in the following trotting conditions: straight hard (SH), straight soft (SS), left and right circle hard (LCH and RCH), left and right circle soft (LCS and RCS). Statistical significance is shown as * (*p* < 0.05) and ** (*p* < 0.01).

The comparisons between the absolute values of the studied locomotion parameters measured under the different assessment conditions are shown in Figure 10 and Figure 11. For IMUs, significantly higher MinDiff_pelvis_ values were found for LCS compared to SH and SS (Figure 10). Regarding AI-MTS, the differences between the various assessment conditions were found in MaxDiff_head_. Higher values for MaxDiff_head_ were found in LCH compared to SH and SS and in LCS compared to SH and SS (Figure 11).

## 4. Discussion

The present study was performed due to the recent development of an AI marker-less motion tracking system (AI-MTS). This system is intended for the assessment of lameness/asymmetry and can be easily used in a clinical setting to aid in the decision-making process [33,34]. In this study, our aim was to compare a commercial IMU system, previously validated and widely used [13,17,18,22,38,39], with this new system (AI-MTS) [33,34,40] in clinically sound horses and in horses with a mild intensity lameness, under different assessment conditions, i.e., a straight line and in right and left circles, on both hard and soft surfaces.

The most significant findings were the following: (1) The number of horses considered sound in the clinical examination was higher compared to the number of asymmetric animals detected by the IMU and AI-MTS systems. (2) A complete agreement between the clinical examination and the two gait assessment systems regarding the affected limb(s) occurred only in a very reduced number of animals because IMUs and AI-MTS detected more asymmetrical limbs than clinical examination. (3) The greatest agreement between IMUs and AI-MTS was found for MinDiff_head_ in SH conditions. MaxDiff_head_ and MaxDiff_pelvis_ showed substantial agreement in circles, particularly towards the right side and on both types of surface types (hard and soft). The lowest agreements were found for MinDiff_pelvis_ in all conditions. (4) Finally, considering the absolute values of the evaluated locomotor parameters, the IMU system presented higher values for MinDiff_head_ (in SH and SS conditions) and for MinDiff_pelvis_ (in SH and LCS conditions) and lower values for MaxDiff_head_ (in the LCS condition). 

The fact that the number of asymmetric horses detected by both gait assessment systems was higher than the number of horses considered lame by clinical examination was an expected finding. Clinical examination, using the human eye and depending on the experience of the examiner, detects more obvious changes in gait, as well as other clinical manifestations that are not detected by gait assessment systems, which are based on an asymmetry or gait deviation that is considered ‘normal’ or ‘perfect’. Mild asymmetries may have gone unnoticed during the clinical examination, particularly when more than one limb was affected and/or when compensatory movements affected the other limbs. In fact, at a subclinical level, the human ability to detect very mild asymmetries may be limited. This idea was supported by the finding that the limb(s) considered lame during the clinical examination were also detected as asymmetric by the two gait analysis systems assessed. However, the number of affected limb(s) was greater with AI-MTS initially, followed by IMUs, compared to the number of limb(s) identified as lame by clinical examination. Moreover, of the horses considered lame on clinical examination (*n* = 11), seven presented lameness in the forelimbs. The detection of very subtle hindlimb lameness is more complex and may go unnoticed, and this fact may partially justify the detection of a greater number of asymmetrical limbs with the IMU and AI-MTS systems compared to clinical examination. This result deserves to be highlighted, since the correct identification of hindlimb lameness by clinical examination is very challenging, mainly because vertical movements of the pelvis and pelvic rotations are highly dependent on factors other than lameness, such as physical anatomy, conformation [41], as well as different movement patterns and hip rotation adaptations [41,42,43,44]. Interestingly, we have also noticed that the previously published cut-off values for the distinction between symmetry and asymmetry by the IMUs [16,45] are less sensitive compared to the ranges adopted in the present study for the classification of asymmetry severity [46,47]. Indeed, when defining asymmetry in a dichotomous way (presence/absence), very mild asymmetries may go under-diagnosed. The thresholds used in this study did not consider breed, age, or sex groups, so it may be interesting to perform gait analysis using these systems in the near future, establishing reference threshold cut-off values tailored for different populations. 

There is much debate about the clinical significance of asymmetries detected by gait assessment systems. As mentioned previously, gait asymmetry does not imply lameness, which is a clinical concept with several different implications, such as a reduction in athletic performance or an impact on welfare during exercise and training. In fact, numerous studies [46,48,49] have shown that 50–72% of the horses considered sound by their owners exhibited gait asymmetry outside normal values when quantified using IMUs. The relevance of these asymmetries to athletic performance is currently unknown. In a population of 33 elite eventing horses in competition [47], authors found that 61% of these animals exhibited locomotor asymmetries. However, the clinical relevance of these asymmetries was not demonstrated, as no differences were found between pre- and post-competition, and the horses were considered ‘fit for competition’ by the veterinary staff. Interesting results were described by Lopes et al. (2018) [50] in endurance horses in competition. Using an IMU system, 21 of the 22 endurance horses evaluated were considered asymmetric, and there was a significant disagreement between these results and the clinical assessment of the competition veterinarians, although this disagreement disappeared when the sensitivity of the IMUs was reduced. Considering these data together, it seems that gait analysis systems are too sensitive, and perhaps the sensitivity should be reduced so it can be used in a competitive setting, but this hypothesis must be analyzed in further detail.

In our case, all horses assessed performed as expected by their owners. However, it should be considered that none of the horses studied were elite athletes. In the near future, it would be interesting to include these locomotor assessments in performance tests and to analyze their changes throughout the competitive season for each individual. There are numerous physiological factors that may influence locomotor asymmetry without being associated with lameness. This idea is supported by data provided by Jansson et al. (2021) [51], who found that horses subjected to a high-energy diet experienced weight gain and exhibited greater locomotor asymmetry compared to those animals subjected to a 36-day dietary restriction.

The greatest agreement between IMUs and AI-MTS was found for MinDiff_head_ in SH conditions. This is possibly due to a greater regularity of the assessment surface, together with easier identification of anatomical landmarks in the case of AI-MTS. In addition, horses have highly symmetrical peak vertical forces, stance duration, and limb impulses during the stance phase when trotting in a straight line [52], which explains the higher level of head agreement in this condition. The lower level of agreement between the two systems for MinDiff_pelvis_ can be explained by the fact that the AI-MTS recorded a lower number of hindlimb strides compared to IMUs, since the pelvis was predominantly visible when the horse trotted away from the camera. This can lead to biases since the resulting asymmetries will be influenced by variations in the underlying data in terms of stride selection. Finally, we observed that the absolute values of MinDiff_head_ (SH and SS) and MinDiff_pelvis_ (SH and LCS) were higher when measured by IMUs compared to AI-MTS, while those of MaxDiff_head_ (LCS) were higher when measured by AI-MTS. These results suggest that IMUs may be more sensitive for detecting impact asymmetries, while AI-MTS for push-off asymmetries. Since these differences were significant only in some assessment conditions, further studies should be conducted to confirm our hypothesis.

## 5. Conclusions

In this study, horses of different breeds were clinically assessed for lameness, and their locomotor asymmetry was analyzed by two different methods: IMUs and AI-MTS. The number of horses considered sound by clinical examination was greater than the number of asymmetrical animals detected by the two different gait analysis systems. However, limb(s) identified as lame by clinical examination were also identified as asymmetrical by both IMU and AI-MTS. A greater number of limbs were identified asymmetrical by AI-MTS and IMUs, suggesting a greater sensitivity of these technologies compared to visual assessment, particularly in the hind limbs, but the different thresholds set for asymmetry might have to be taken into consideration. The agreement between both IMUs and AI-MTS was greater for straight lines and hard surfaces, particularly for MinDiff_head_. The lowest agreement was found for MinDiff_pelvis_, reflecting the difficulties in assessing asymmetry in the hind limbs. Some degree of asymmetry is likely to be clinically relevant, suggesting the need for regular assessment. In the future, the introduction of these measures in training horses, together with those derived from physiological adaptations to exercise tests (such as heart rate and blood lactate accumulation), is strongly recommended. 

## Figures and Tables

**Table 1:** Lameness scoring is conducted based on clinical lameness examination and assessment of outcomes from the inertial measurement unit (IMU) system, using two different cut-off values (in millimeters, to distinguish between symmetric and asymmetric horses, as well as for severity assessment), and an artificial intelligence marker-less motion tracking system (AI-MTS). The results of IMU and AI-MTS assessments in horses, conducted while trotting on a straight line and hard surface, are presented.

Horse	AAEP Grade	IMUs		AI-MTS
		Millimeters Cut-Off	Severity	
1	RF: 2/5	Asymmetric (FL, HL)	RF: moderate impact, very mild push-off; RH: very mild impact	RF: mild impact, mild push off; RH: very mild impact
2	Sound	Symmetric	LF: very mild impact	LF: very mild impact
3	RF: 2/5	Asymmetric (FL)	RF: very mild impact, very mild push-off; LH: very mild push-off	RF: mild impact, very mild push-off; LH: very mild push-off
4	Sound	Asymmetric (HL)	LH: very mild impact, very mild push-off	Symmetric
5	LF: 1/5; LH: 2/5	Asymmetric (FL, HL)	LF: mild impact, mild push-off; LH: very mild impact, mild push-off	LF: mild impact, mild push-off; LH: mild push-off
6	Sound	Symmetric	LF: very mild impact, very mild push-off; RH: mild push-off	LF: very mild impact; RH: mild push-off
7	RF: 1/5	Asymmetric (FL)	RF: very mild impact; LF: very mild push-off; LH: very mild impact, very mild push-off	RF: mild impact; LF: very mild push- off; RH: very mild impact; LH: very mild push-off
8	Sound	Symmetric	RF: very mild push-off	RF: very mild push-off
9	LF: 1/5	Asymmetric (FL, HL)	LF: mild impact; LH: very mild impact, very mild push-off	LF: very mild impact
10	RF: 2/5	Asymmetric (FL)	RF: mild impact; RH: very mild push-off	RF: very mild impact; RH: very mild impact
11	LH: 1/5	Asymmetric (HL)	LH: very mild impact., very mild push-off	LH: very mild push-off
12	Sound	Asymmetric (HL)	LF: very mild impact; RH: very mild impact	LF: very mild impact
13	LF: 1/5	Asymmetric (FL)	LF: mild impact	LF: very mild impact, very mild push-off; RH: very mild impact
14	LH: 1/5	Asymmetric (HL)	LH: very mild impact, mild push-off	LH: very mild impact, very mild push-off
15	Sound	Asymmetric (FL)	RF: very mild impact	RH: very mild impact
16	RF: 1/5	Asymmetric (FL)	RF: mild impact, mild push-off	RF: very mild impact, very mild push-off
17	LF: 1/1	Symmetric	LF: very mild impact; LH: very mild push-off	LF: very mild impact; LH: very mild push-off
18	Sound	Symmetric	RF: very mild impact; LF: very mild push-off; LH: very mild impact	RF: very mild impact; RH: very mild push-off
19	Sound	Asymmetric (HL)	LF: very mild impact; RH: very mild push-off; LH: very mild impact	RH: very mild push-off
20	Sound	Symmetric	LF: very mild impact; RH: mild push-off	RH: very mild push-off; LH: very mild impact

FL = forelimb, HL = hindlimb, RF = right forelimb, LF = left forelimb, RH = right hindlimb, LH = left hindlimb.

**Table 2:** Cohen’s K coefficients of agreement between the inertial measurement unit (IMU) system and an artificial intelligence motion tracking system (AI-MTS) in the following assessment conditions: straight hard (SH), straight soft (SS), left circle hard (LCH), left circle soft (LCS), right circle hard (RCH), and right circle soft (RCS).

Locomotion Parameters	SH	SS	LCH	LCS	RCH	RCS
MinDiff_head_	0.80	0.65	0.58	0.53	0.67	0.60
MaxDiff_head_	0.55	0.25	0.54	0.54	0.74	0.77
MinDiff_pelvis_	0.12	0.08	0.35	0.17	0.47	0.59
MaxDiff_pelvis_	0.50	0.51	0.49	0.68	0.69	0.63

**Table 3:** Number of valid strides recorded by the inertial measurement unit (IMU) system and the artificial intelligence motion tracking system (AI-MTS) in the different assessment conditions (SH, SS, LCH, RCH, LCS, RCS). Data are expressed as mean ± SD. Data in rows with different superscript letters are significantly different from each other (*p* < 0.05).

Assessment Condition	IMUs	AI-MTS	
		Forelimbs	Hindlimbs
Straight hard (SH)	23 ± 7 ^b^	34 ± 9 ^a^	21 ± 7 ^b^
Straight soft (SS)	21 ± 6 ^a^	25 ± 6 ^a^	16 ± 4 ^b^
Left circle hard (LCH)	28 ± 11 ^c^	44 ± 14 ^a^	37 ± 11 ^b^
Left circle soft (LCS)	30 ± 9 ^b^	40 ± 16 ^a^	34 ± 11 ^b^
Right circle hard (RCH)	29 ± 12 ^c^	48 ± 9 ^a^	36 ± 8 ^b^
Right circle soft (RCS)	29 ± 12 ^b^	42 ± 15 ^a^	35 ± 10 ^b^

## Data Availability

The data presented in the current study are available from the corresponding author upon reasonable request.
